# Chrysomelinae species (Coleoptera, Chrysomelidae) and new biological data from Rio de Janeiro, Brazil

**DOI:** 10.3897/zookeys.720.13963

**Published:** 2017-12-11

**Authors:** Vivian Flinte, André Abejanella, Mauro Daccordi, Ricardo F. Monteiro, Margarete Valverde Macedo

**Affiliations:** 1 Av. Carlos Chagas Filho, 373. CCS, IB, Laboratório de Ecologia de Insetos, Universidade Federal do Rio de Janeiro, Ilha do Fundão, CEP 21941-590, Rio de Janeiro, RJ, Brazil; 2 Museo Civico di Storia Naturale, Lungadige Porta Vittoria 9, 37129, Verona, Italy

**Keywords:** Atlantic forest, biodiversity, host plant, *Platyphora*, seasonality, viviparity

## Abstract

Chrysomelinae is one of the largest subfamilies in Chrysomelidae, yet much basic information remains unknown for Neotropical species. The present study aims to compile the first regional list of Chrysomelinae for the State of Rio de Janeiro, Brazil, and assemble natural history traits obtained from our fieldwork from 2005 to 2010 in Serra dos Órgãos National Park, a mountainous area of Atlantic forest. The species list was compiled from data from field work, collections, and literature, and recorded a total of 100 species, belonging to 21 genera in one tribe (Chrysomelini) and three subtribes: Chrysolinina (91 species), Chrysomelina (eight species) and Entomoscelina (one species). Of these, 91 species are new records for the state. Serra dos Órgaõs National Park holds records of 43 species, with *Platyphora* being the most species-rich genus, and Solanaceae the most common host plant family. Some new records of reproductive mode (larviparous vs. oviparous) and larval behavior are also given. These Brazil Chrysomelinae species exhibited a clear seasonal pattern, with more species recorded in the hot and rainy season from October to January, and considerably fewer species from June to August, during the drier and colder months. The fraction of new records in comparison with published species and natural history information illustrates how little we know of Chrysomelinae in the state and in the country.

## Introduction

Chrysomelinae is the fifth largest subfamily of Chrysomelidae, after Galerucinae, Eumolpinae, Cassidinae and Cryptocephalinae (Reid 2006), with 3,000 species and 132 genera (Daccordi 1994, 1996, Riley et al. 2002), but these numbers vary among authors (see Seeno and Wilcox 1982, Reid 1995). Two tribes are generally recognized: Timarchini, which is monogeneric with *Timarcha* Latreille (ca. 100 species); and Chrysomelini containing the remainder (Seeno and Wilcox 1982, Daccordi 1994). However, there are still many problems concerning Chrysomelinae taxonomy. Daccordi (1996) listed 38 genera for the Neotropical region, out of which 31 are exclusive to the area. Some of the main contributions for Neotropical Chrysomelinae taxonomy and cataloguing are those by Jan Bechyně (e.g. 1954, 1958, 1980), which include many species descriptions and some regional lists, and, more recently, a key to the genera in Costa Rica by Wills Flowers (2004), modified from Bechyně and Springlova de Bechyně (1965). Both larvae and adults normally feed on leaves of the same host plant species and species tend to be monophagous or to feed on a narrow group of related plant species (Jolivet 1988). The same author pointed out that host plants are known for nearly 40% of Chrysomelinae genera, and data are largely lacking for tropical species. In the Neotropical area, Chrysomelinae are frequently associated with Solanaceae, Asteraceae, Apocynaceae, and Zygophyllaceae (Jolivet and Hawkeswood 1995). However, knowledge of Chrysomelinae biology is rare in this region, remaining so two decades after being underlined by Jolivet (1997).

Except for some ecological studies and species records confined to entomological collections, no list of species exists for the subfamily in the State of Rio de Janeiro or even in Brazil. Since we have conducted extensive research in a protected area in the state and have accumulated considerable biological information on Chrysomelidae species, our aim here is to compile the first regional list of Chrysomelinae in Brazil, and to assemble natural history traits for the species found in Serra dos Órgãos National Park, State of Rio de Janeiro.

## Materials and methods

For Chrysomelinae species list compilation for Brazil, four national collections were examined: Coleção entomológica do Laboratório de Ecologia de Insetos / Universidade Federal do Rio de Janeiro, Rio de Janeiro (**CLEI**); Museu Nacional / Universidade Federal do Rio de Janeiro, Rio de Janeiro (**MNRJ**), except species from Itatiaia; Coleção entomológica da Fundação Instituto Oswaldo Cruz, Rio de Janeiro (**CEIOC**); and Museu Paraense Emílio Goeldi, Belém (**MPEG**). The digital collection of the Museo del Instituto de Zoologia Agricola, Universidad Central de Venezuela (MIZA) was also consulted. Finally, the literature was searched for additional records. These records are indicated in Table 1. Location is given by the municipality within the State of Rio de Janeiro, which comprises 43,696 km² and represents less than 1% of the country’s area. Taxonomy follows Daccordi (1994) and Seeno and Wilcox (1982).

**Table 1.** List of Chrysomelinae species. Chrysomelinae species from the State of Rio de Janeiro, indicating the municipality of the record and specific location, when available. Numbers indicate the source of information (see footnote below table). SONP = Serra dos Órgãos National Park; INP = Itatiaia National Park.

Species	Location
**Chrysomelini: Chrysolinina** – **15 genera and 91 species**	
*Calligrapha polyspila* (Germar, 1821) (Fig. 1A)	Angra dos Reis^3^, Itatiaia^3^, Paraty (Pedra Branca)^1^, Resende^3^, Teresópolis^3^ (SONP^1^)
*Cosmogramma decora* Stål, 1859	Itatiaia (INP)^1^
*Cosmogramma fulvocincta* Stål, 1859	Itatiaia^3^
*Cosmogramma wygodzinskyi* Bechyně, 1948	Itatiaia^4^
*Cryptostetha hieroglyphica* Lucas, 1857	Itatiaia^3^ (INP^1^)
*Cryptostetha notatifrons* Stål, 1863	Itatiaia^3^
*Deuterocampta achardi* Bechyně, 1944	Mendes^4^
*Deuterocampta cruxnigra* Stål, 1859	Angra dos Reis^3^
*Deuterocampta fallax* Bechyně, 1950	Itaboraí^2^, Rio de Janeiro (Gávea^4^, Tijuca^2^)
*Deuterocampta humeralis* Bechyně, 1944	Petrópolis (SONP)^3^
*Deuterocampta leucomelaena* (Perty, 1832)	Itatiaia^3,4^ (INP^1^)
*Deuterocampta pustulicollis* Stål, 1859	Macaé^2,4^
*Deuterocampta sedula* Stål, 1859 (Fig. 1B)	Teresópolis^2^ (SONP^1^)
*Deuterocampta semistriata* (Fabricius, 1775)	Petrópolis (SONP)^4^, Rio de Janeiro (Rio de Janeiro^4^, Corcovado^3^)
*Deuterocampta stauroptera* (Wiedmann, 1821)	Rio de Janeiro (Botafogo^4^, Corcovado^3^, Gávea^4^, Rio de Janeiro^3^, Tijuca^4^)
*Deuterocampta undulata* Bechyně, 1950	Rio de Janeiro^4^
*Deuterocampta vittulosa* Bechyně,1944	Rio de Janeiro (Engenho de Dentro^4^)
*Dorysterna cruentata* (Baly, 1858)	Cambuci (Funil)^3^
*Dorysterna dorsosignata* (Stål, 1857)	Itatiaia (INP)^1^, Rio de Janeiro (Corcovado^2^, Rio de Janeiro^2^)
*Dorysterna riopardensis* Bechyně, 1948	Nova Friburgo^2^
*Dorysterna salvatori* Bechyně, 1948	Teresópolis (SONP)^1^
*Elytrosphaera breviuscula* Stål, 1858	Grande Rio (Baixada fluminense^4^)
*Elytrosphaera lahtivirtai* Bechyně, 1951	Itatiaia (INP^1^)
*Elytrosphaera noverca* Stål, 1858	Teresópolis (SONP)^1^
*Elytrosphaera xanthopyga* Stål, 1858 (Fig. 1C)	Itatiaia^1,3^, Resende^3^, Teresópolis^2,3^ (SONP^1^)
*Eugonycha bryanti* Bechyně, 1946	Rio de Janeiro^4^
*Gavirga subaenea* Bechyně, 1946	Itatiaia^4^
*Grammodesma elongata* Bechyně, 1952	Itatiaia (INP)^8^
*Grammodesma luridipennis* (Baly, 1859)	Itatiaia (INP)^8^
*Grammodesma obliqua* (Stål, 1859)	Itatiaia^3,4^ (PNI)^8^
*Grammodesma rubroaenea* (Stål, 1859) (Fig. 1D)	Teresópolis (SONP)^1^
*Grammodesma stulta* (Stål, 1859)	Rio de Janeiro (Corcovado^2^, Rio de Janeiro^4^, Tijuca^2^)
*Metastyla insignis* Achard, 1923	Rio de Janeiro (Corcovado^2,4^, Rio de Janeiro^3^, Tijuca^2^)
*Monocampta crucigera* (Sahlberg, 1823)	Angra dos Reis^3^, Itatiaia (Itatiaia^3^, Penedo^3^), Rio de Janeiro (Alto da Boa Vista^3^, Corcovado^3^, Tijuca^3,4^), Teresópolis^2^ (SONP^1^)
*Platyphora acuminata* (Olivier, 1790)	Itatiaia^3^
*Platyphora angulata* Stål, 1858	Rio de Janeiro^5^
*Platyphora axillaris* (Germar, 1824) (Fig. 1E)	Angra dos Reis^3^, Itatiaia^3^, Nova Friburgo^3^, Rio de Janeiro (Gávea^3^, Tijuca^3^), Silva Jardim^1^, Teresópolis^2,3^ (SONP^1,10^), Guapimirim (SONP)^1^, Três Rios^3^
*Platyphora biforis* (Germar, 1824)	Itatiaia^3^, Laje do Muriaé^3^, Rio de Janeiro^2^
*Platyphora bigata* (Germar, 1824) (Fig. 1F)	Teresópolis^3^ (SONP)^1^
*Platyphora bullata* (Stål, 1858)	Nova Friburgo^2^
*Platyphora cincta* (Germar, 1821)	Itatiaia^3^, Teresópolis (SONP)^3^
*Platyphora congener* (Stål, 1858) (Fig. 1G)	Nova Iguaçu (ReBio do Tinguá^3^), Rio de Janeiro (Tijuca^3^), Teresópolis (SONP)^1^
*Platyphora conviva* (Stål, 1858)	Itatiaia^3^ (INP^1^)
*Platyphora curticollis* (Stål, 1857) (Fig. 1H)	Teresópolis (SONP)^1^
*Platyphora dejeani* (Germar, 1824) (Fig. 1I)	Casimiro de Abreu (ReBio União)^1^, Itatiaia^3^, Nova Iguaçu (ReBio do Tinguá)^1^, Petrópolis^1^, Rio de Janeiro (Corcovado^3^, Tijuca^3^), Teresópolis^2,3^ (SONP)^1^
*Platyphora difficilis* (Stål, 1859) (Fig. 1J)	Teresópolis (SONP)^1^
*Platyphora dilaticollis* (Stål, 1858)	Cambuci (Funil)^3^, Itatiaia^3^, Teresópolis (SONP)^1^
*Platyphora fasciatomaculata* (Stål, 1857) (Fig. 1K)	Itatiaia (INP)^1^, Teresópolis (SONP)^1^
*Platyphora fervida* (Fabricius, 1775) (Fig. 1L)	Itatiaia^3^, Teresópolis^2^ (SONP^1,9^)
*Platyphora figurata* (Germar, 1824)	Angra dos Reis^3^, Rio de Janeiro^3^
*Platyphora flavovittata* (Stål, 1858) (Fig. 1M)	Itatiaia^3^ (INP^1^), Teresópolis (SONP)^1^
*Platyphora fraterna* (Stål, 1857) (Fig. 1N)	Teresópolis (SONP)^1^
*Platyphora histrio* (Olivier, 1807)	Angra dos Reis^3^, Itatiaia^3^, Rio de Janeiro (Rio de Janeiro^2^, Corcovado^3^),
*Platyphora irrorata* (Stål, 1857)	Itatiaia^3^, Rio de Janeiro (Corcovado^3^, Rio de Janeiro^3^)
*Platyphora itatiayensis* (Bechyně, 1950) (Fig. 1O)	Itatiaia^3^, Teresópolis (SONP)^1^
*Platyphora jucunda* (Stål, 1857) (Fig. 1P)	Itatiaia^3^, Teresópolis (SONP)^1^
*Platyphora langsdorfi* (Germar, 1824) (Fig. 1Q)	Teresópolis (SONP)^1^
*Platyphora pardalina* (Stål, 1858)	Itatiaia^3^
*Platyphora pastica* (Germar, 1824) (Fig. 1R)	Angra dos Reis^3^, Itatiaia^3^, Rio de Janeiro (Alto da Boa Vista^3^), Teresópolis (SONP)^1^
*Platyphora pervicax* (Stål, 1859)	Itatiaia^3^
*Platyphora princeps* Gray, 1832	Itatiaia^3^
*Platyphora reticulata* (Fabricius, 1787)	Itatiaia^3^, Teresópolis (SONP)^3^
*Platyphora semiviridis* Jacoby, 1903	Itatiaia^3^, Resende^6^
*Platyphora signiceps* (Stål, 1857)	Itatiaia^3^, Petrópolis (SONP)^3^
*Platyphora* sp.	Itatiaia (INP)^1^
*Platyphora strigilata* (Stål, 1859)	Itatiaia^3^ (INP^1^)
*Platyphora tesselata* (Olivier, 1807)	Teresópolis (SONP)^3^
*Platyphora variolaris* (Stål, 1859)	Nova Friburgo^2^
*Platyphora vidanoi* Daccordi,1993 (Fig. 1S)	Itatiaia^3^ (INP^1^), Teresópolis (SONP)^1^
*Platyphora vigintiunopunctata* (Chevrolat, 1831)	Itatiaia^3^, Teresópolis (SONP)^2^
*Platyphora zikani* (Bechyně,1950) (Fig. 1T)	Teresópolis (SONP)^1^
*Platyphora zonata* (Germar, 1824) (Fig. 1U)	Macaé (Parque Nacional da Restinga de Jurubatiba)^1^, Itatiaia^3^, Teresópolis (SONP)^1^
*Stilodes flavosignata* (Stål, 1859)	Nova Friburgo^2^, Rio de Janeiro (Rio de Janeiro^2^, Corcovado^3^), Teresópolis (SONP)^1^
*Stilodes jocosa* (Stål, 1859)	Rio de Janeiro (Corcovado^2,4^)
*Stilodes nigriventris* (Germar, 1824)	Itaguaí^2^, Macaé (Restinga de Jurubatiba)^1^, Rio de Janeiro (Corcovado^2,4^)
*Stilodes peltasta* (Stål, 1865)	Rio de Janeiro (Corcovado^2^)
*Stilodes* sp. 1	Teresópolis (SONP)^1,9^
*Stilodes* sp. 2	Teresópolis (SONP)^1^
*Stilodes thetis* Stål, 1860 (Fig. 1V)	Itatiaia (INP)^1^, Teresópolis (SONP)^1^
*Stilodes trimaculicollis* Stål, 1859	Rio de Janeiro (Rio de Janeiro^3^, Corcovado^2^), Teresópolis (SONP)^1^
Stilodes (Eustilodes) cordata Achard, 1923	Rio de Janeiro^4^, Teresópolis (SONP)^1^
Stilodes (Eustilodes) cornuta (Bechyně, 1947)	Itatiaia^3^
Stilodes (Eustilodes) denticeps (Stål, 1860)	Macaé^4^
Stilodes (Grammomades) impuncticollis (Stål, 1859) (Fig. 1W)	Itatiaia^3^, Laje do Muriaé^3^, Teresópolis^2^ (SONP^1,9^)
Stilodes (Isostilodes) bisbilineata Stål, 1859	Itatiaia^3^
*Trichomela notaticollis* (Stål, 1858)	Itatiaia^3^, Teresópolis (SONP)^3^
*Trichomela xantholoma* (Stål, 1857) (Fig. 1X)	Teresópolis (SONP)^1^
*Zygogramma appendiculata* Stål, 1859 (Fig. 1Y)	Teresópolis (SONP)^1^
*Zygogramma novemstriata* Stål, 1859	Angra dos Reis^3^
Zygogramma (Tritaenia) mendesi Bechyně, 1948	Itatiaia^3,4^, Resende^3^
Zygogramma (Tritaenia) virgata (Stål, 1859)	Rio de Janeiro (Tijuca^2^)
**Chrysomelini: Chrysomelina** – **5 genera and 8 species**	
*Lioplacis meridionalis* Bechyně, 1948	Itatiaia (INP)^1^
*Phaedon confinis* Klug, 1829	Angra dos Reis^3^, Itatiaia^3^
*Phaedon consimilis* Stål, 1860	Rio de Janeiro (Manguinhos^3^)
*Phaedon pertinax* Stål, 1860	Nova Friburgo^4^, Itatiaia^3^, Resende^3^, Rio de Janeiro (Manguinhos^3^)
*Pixis clavigera* Stål, 1860	Rio de Janeiro (Corcovado^2^)
*Pixis columbina* Stål, 1860	Itatiaia^7^, Teresópolis (SONP)^1^
*Plagiodera gounelli* Achard, 1925	Rio de Janeiro (Corcovado^2^, Tijuca^2^)
*Trochalonota badia* (Germar, 1824)	Rio de Janeiro (Anil^2^, Corcovado^2^, Tijuca^3^)
**Chrysomelini: Entomoscelina** – **1 genus and 1 species**	
*Microtheca ochroloma* Stål, 1860	Rio de Janeiro (Deodoro^2^, Rio de Janeiro^2^)

^1^ CLEI; ^2^ MNRJ; ^3^ CEIOC; ^4^ MIZA, ^5^ MPEG; ^6^ Olckers 1998; ^7^ Bechyně 1958; ^8^ Sampaio and Monné 2016; ^9^ Flinte et al. 2009b; ^10^ Flinte et al. 2015.

For documentation of species’ natural history and host plants, data assembled from field expeditions during different research projects conducted at Serra dos Órgãos National Park (22°26'56"S; 42°59'5"W), State of Rio de Janeiro, between 2005 and 2010 was used. The duration, months and number of participants of field expeditions per year are as follows: 2005 (1 or 2 days every month, 3 to 5 collectors); 2006 (2 to 4 days every month, 3 to 5 collectors); 2007 (2 to 4 days every month, 3 to 5 collectors); 2008 (1 or 2 days every month, 2 or 3 collectors); 2009 (1 or 2 days every month, 2 or 3 collectors); 2010 (1 or 2 days every month, 1 or 2 collectors).

### Study Site

The park covers an area of 20,024 ha of well-preserved Atlantic Rain Forest (see Veloso et al. 1991 for more on local vegetation) and is located ca. 100 km from Rio de Janeiro, in a mountainous area ranging from 80 m to 2263 m elevation. The climate is tropical, with a colder drier season from May to August, and a rainy warmer period from November to February (Flinte et al. 2009b). Mean annual temperature is around 18 °C, maximum of 38 °C and minimum of 0 °C. Annual precipitation varies between 1250 and 1500 mm (Flinte et al. 2008).

### Species study and collection

Species were sampled by a combination of manual collecting, sweep nets and malaise traps, during the conduction of other projects with Chrysomelidae in the park. When a species was initially found in the field, individuals in as many different developmental stages as possible were brought to the laboratory and reared in plastic containers for host plant confirmation and observations on behavior and biology. In an attempt to describe species seasonal distribution in the area, considering data on labels of specimens from all collections, we recorded the different months on which they were collected and summed the number of species per month (independent of year).

### Identification and vouchers

Chrysomelinae species were identified by Mauro Daccordi. Solanaceae host plants were identified by Lucia d’Ávila Freire de Carvalho (Jardim Botânico do Rio de Janeiro) and Luciano Bianchetti (Embrapa/Brasília), Asteraceae by Roberto Lourenço Esteves (Universidade do Estado do Rio de Janeiro), Convolvulaceae by Rosângela Simão-Bianchini (Herbário SP - Instituto de Botânica) and Malvaceae by Massimo Bovini (Jardim Botânico do Rio de Janeiro). Thiago Marinho Alvarenga (Universidade de Campinas) identified parasitoids. Species collected at Serra dos Órgãos National Park are deposited at CLEI-UFRJ, Rio de Janeiro, Brazil.

## Results and discussion

### General patterns of richness and distribution

The Chrysomelinae Neotropical fauna is thought to comprise ca. 38 genera (Daccordi 1996) and 1,020 species (Blackwelder 1944), but these are outdated numbers and no such information could be found specifically for Brazil. One hundred species occurring in the State of Rio de Janeiro were recorded, belonging to 21 genera in one tribe (Chrysomelini) and three subtribes: Chrysolinina, Chrysomelina and Entomoscelina (Table 1). Chrysolinina was represented by 91 species, followed by Chrysomelina with eight and Entomoscelina with only one species (Table 1). According to Daccordi (1996), there are many endemic Chrysolinina and Chrysomelina taxa in the Neotropical region, where they reach their maximum diversity. Only nine species are from previously published sources, the other 91 species we found are new records for the state. The genus with most species records was *Platyphora* (n = 39) representing 42.4% of Chrysolinina found and 40% of total species records, followed by *Stilodes* (n = 13, 12.9% of all species recorded) and *Deuterocampta* (n = 11, 10.9%), genera restricted to the Neotropical region (Daccordi 1996). Indeed, *Platyphora* is the most species-rich genus in South America (Daccordi 1994), with approximately 500 species (Chaboo et al. 2014 and references therein).

The findings presented here also revealed a high diversity of species and genera, typical for the Atlantic rain forest, in comparison to other studies in South and Central America. Flowers (2004) documented 67 species in 11 genera for Costa Rica and, similar to our work, *Platyphora* and *Stilodes* were the most species-rich genera. During a six-year field study in a Mexican state, 47 species and eight genera were found; *Leptinotarsa*, *Calligrapha* and *Zygogramma* were the genera with most species records (Burgos-Solorio and Anaya-Rosales 2004). Chaboo and Flowers (2015) found 158 species and 18 genera for Peru, based on species catalogues.

Species were recorded from only 17 (18.5%) of the state’s municipalities, and 62 species were known from only one location (Table 1). We found a similar pattern in an inventory of Cassidinae for the same state (Flinte et al. 2009a), with most records concentrated near the city of Rio de Janeiro and in large protected areas, such as Petrópolis and Teresópolis (Serra dos Órgãos National Park) and Itatiaia (Itatiaia National Park). The high number of single locality records is probably due more to sampling effort than to endemism, considering that species normally are not very abundant and are more easily collected manually than with traps.

### Biology and ecology of Chrysomelinae at Parque Nacional da Serra dos Órgãos

**Figure 1.**
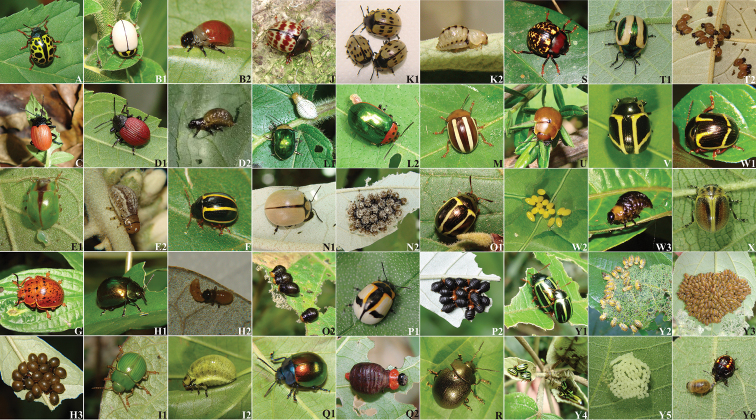
Chrysomelinae species in Rio de Janeiro. Some Chrysomelinae species occurring in Serra dos Órgãos National Park, State of Rio de Janeiro, Brazil. *Calligrapha polyspila* (**A**); *Deuterocampta sedula* adult (**B1**) and larva (**B2**); *Elytrosphaera xanthopyga* (**C**); *Grammodesma rubroaenea* adult (D1) and larva (**D2**); *Platyphora axillaris* adult (**E1**) and larva (**E2**); *Platyphora bigata* (**F**); *Platyphora congener* (**G**); *Platyphora curticollis* adult (**H1**), larval cannibalism (**H2**) and larval aggregation (**H3**); *Platyphora dejeani* adult (**I1**) and larva (**I2**) *Calligrapha polyspila* (*Platyphora difficilis*) (**J**); *Platyphora fasciatomaculata* adult (**K1**) and larva (**K2**); *Platyphora fervida* yellow-pronotum adult and larva (**L1**) and red-pronotum female ovipositing (**L2**); *Platyphora flavovittata* (M); *Platyphora fraterna* adult (**N1**) and larval aggregation (**N2**); *Platyphora itatiayensis* adult (**O1**) and larvae (**O2**); *Platyphora jucunda* adult (**P1**) and larval aggregation (**P2**); *Platyphora langsdorfi* adult (**Q1**) and larva (**Q2**); *Platyphora pastica* (**R**) *Platyphora vidanoi* (**S**); *Platyphora zikani* adult (**T1**) and young larvae (**T2**); *Platyphora zonata* (**U**); *Stilodes thetis* (**V**); Stilodes (Grammomades) impuncticollis adult (**W1**), eggs (**W2**) and larva (**W3**); *Trichomela xantholoma* (**X**); *Zygogramma appendiculata* polymorphic adults in copula (**Y1**), larvae feeding (**Y2**), larval cycloalexy (**Y3**), adult aggregation (**Y4**), egg mass (**Y5**), larva attacked by hemipteran nymph (**Y6**).

A total of 43 species were recorded from Serra dos Órgaõs National Park (Table 1, under SONP; Figure 1), all Chrysomelini, 42 occurring within the subtribe Chrysolinina and only one from Chrysomelina (*Pixis columbina*). Within Chrysolinina, *Platyphora* was the genus with most species records (23 species) out of the 10 genera found, followed by *Stilodes* (7) and *Deuterocampta* (3), much like the pattern found generally over the state (Table 1). Species showed an enormous variation in color. Adult polymorphism expressed by variation in pronotum color was observed in *P. fervida*, (Fig. 1–L1, L2), while the degree of fusion in stripes on the elytra varied greatly among individuals in *Zygogramma appendiculata* (Fig. 1–Y1). Other species, such as *Platyphora axillaris* (Fig. 1–E1), *P. dejeani* (Fig. 1–I1) and *P. fraterna* (Fig. 1–N1), displayed strikingly similar coloration to the leaves of their host plant, while other species including *P. congener* (Fig. 1G), *Calligrapha polyspila* (Fig. 1A) and *Elytrosphaera xanthopyga* (Fig. 1C) were highly conspicuous to the human eye.

**Figure 2.**
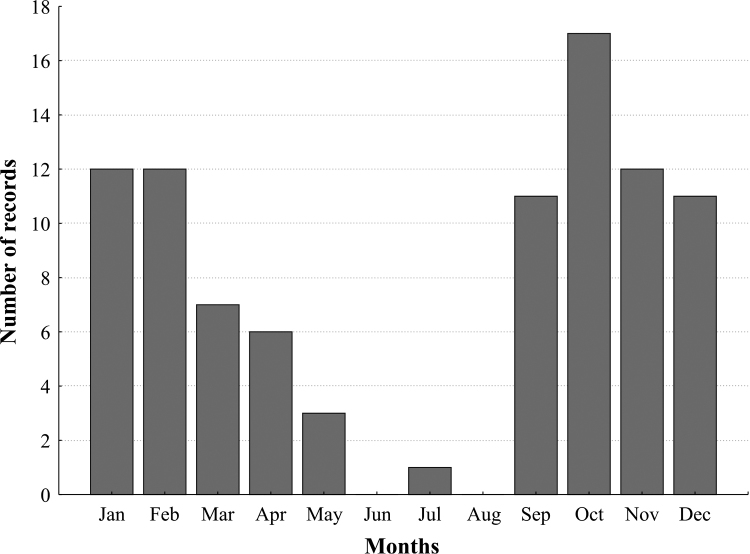
Seasonal distribution of Chrysomelinae. Number of Chrysomelinae species recorded on each month, obtained for 40 species from collections and fieldwork, in Serra dos Órgãos National Park, southeast Brazil.

The subfamily in SONP exhibited a clear seasonal pattern (Fig. 2), with more species recorded in the hot rainy season, from October to January, than during the drier and colder months, between June and August. This seasonal pattern is well-established for the family Chrysomelidae in the area, with annual variation in temperature and precipitation and effects on host plant phenology being likely the main drivers of the temporal dynamics in these beetles (Flinte et al. 2009b, 2011, 2015). This is particularly so because many of the records were made at altitudes above 1000 m, where the pattern normally more closely resembles that found in the subtropical zone (e.g. Medeiros and Vasconcellos-Neto 1994, Nogueira-de-Sá et al. 2004). However, the present results are, to our knowledge, the first to examine the seasonal pattern for such a large number of Chrysomelinae species in a single area. Ideally, a standardized collecting effort across the year would better describe the seasonal differences we observe here. However, as we have conducted research in the area over many years, doing the same surveys at least once a month every year, we are confident that this represents the seasonal pattern of chrysomeline species occurrence in the area. Moreover, the Chrysomelinae species which were intensively studied over the year, *Platyphora axillaris* (Flinte et al. 2015), *P. fervida* and Stilodes (Grammomades) impuncticollis (Flinte et al. 2009b) exhibited the same low densities during the drier and colder months.

Host plant and/or biological information were found for almost half of the species (n = 16) (Table 2) that we (VF, AA, MVM, RFM) collected in the park (n = 35). Solanaceae was the most common host plant family, followed by Convolvulaceae, Asteraceae, Malvaceae and Apocynaceae. As expected, this is a pattern that reflects *Platyphora* preference for Solanaceae (Jolivet and Hawkeswood 1995, Chaboo et al. 2014 and references therein). Intense host plant defoliation was observed in Stilodes (Grammomades) impuncticollis (Fig. 1–W1, W2, W3) on *Capsicum mirabile* (Solanaceae), *Platyphora fraterna* (Fig. 1–N1, N2) on *Solanum swartzianum* (Solanaceae) and *Zygogramma appendiculata* (Fig. 1–Y1, Y2, Y3) on *Callianthe rufinerva* (Malvaceae).

**Table 2.** Ecological data on Chrysomelinae species. Species at Serra dos Órgãos National Park with host plant record and/or biological data obtained from our research at the area. Published records are indicated by numbers (see footnote for references).

Species	Host plant family	Host plant species	Reproduction	Larvae
*Calligrapha polyspila*	Malvaceae ^1^	?	oviparous^1^	?
*Deuterocampta sedula*	?	?	?	solitary
*Grammodesma rubroaenea*	Asteraceae	?	oviparous	solitary
*Platyphora axillaris*	Solanaceae ^2^	*Solanum scuticum* ^2^	larviparous^2^	solitary
*Platyphora curticollis*	Solanaceae	*Solanum swartzianum*	larviparous	aggregated
*Platyphora dejeani*	Convolvulaceae	*Ipomoea philomega*	oviparous	solitary
*Platyphora fervida*	Solanaceae ^3^	*Solanum lhotskyanum* ^3^	larviparous	solitary
*Platyphora flavovittata*	Apocynaceae	?	oviparous	?
*Platyphora fraterna*	Solanaceae	*Solanum swartzianum*	larviparous	aggregated
*Platyphora itatiayensis*	Solanaceae	*Solanum megalochiton*	larviparous	aggregated
*Platyphora jucunda*	Solanaceae ^4^	*Solanum swartzianum*	larviparous	aggregated
*Platyphora langsdorfi*	Convolvulaceae ^5^	*Ipomoea philomega*	oviparous	solitary
*Platyphora zikani*	Solanaceae	*Solanum swartzianum*	larviparous	aggregated
Stilodes (Grammomades) impuncticollis	Solanaceae ^3^	*Capsicum mirabile* ^3^	oviparous	solitary
*Stilodes* sp. 1	Asteraceae	*Baccharis stylosa*	?	?
*Zygogramma appendiculata*	Malvaceae	*Callianthe regnelli*, *Callianthe rufinerva*	oviparous	aggregated

^1^ Grissell et al. 1987; ^2^ Flinte et al. 2015; ^3^ Flinte et al. 2009b; ^4^ Olckers 2000; ^5^ Jolivet and Hawkeswood 1995.

Maternal care was not recorded for any species in this study, although subsocial behavior is known in the subfamily for several species, including some *Doryphora* and *Prosicela* species (Windsor et al. 2013, Chaboo et al. 2014). Other interesting behavioral defenses were recorded, including larval cycloalexy in *Z. appendiculata* (Fig. 1–Y3) and *P. curticollis* (Fig. 1–H3), a defensive behavior of gregarious circular formation at rest (Jolivet et al. 1990, Vanconcellos-Neto and Jolivet 1994, Dury et al. 2014). Additionally, we recorded larvae of *P. fraterna* (Fig. 1–N2) attaching trichomes from *Solanum* host plant leaves to hairs on their backs, a behavior already described in *P. zonata* (Bernardi and Scivittaro 1991), which may contribute to larval camouflage. Larval aggregations were observed in many species (Table 2), but also for adults of *Z. appendiculata* on young folded leaves in the field (Fig. 1–Y4). Larval gregarious behavior may serve to reduce individual risk against small invertebrate parasitoids and predators, and promote defense against larger predators through the cumulative effect of individuals’ toxins (Grégoire 1988). Thanatosis (“feigning death”) was observed in adults of *P. axillaris* (Fig. 1–E1), *P. fervida* (Fig. 1–L1, L2), and *P. fraterna* (Fig. 1–N1), and both in adults and larvae of Stilodes (Grammomades) impuncticollis (Fig. 1–W1, W3).

Seven oviparous and seven larviparous species were found, most being new records of reproductive biology (Table 2). Chrysomelinae is the subfamily of leaf beetles with the most diversity in reproductive biology, containing oviparous, ovoviviparous and viviparous species (Bontems 1988), sometimes in the same genus, and also different levels of social behavior (Chaboo et al. 2014). The last two types of development may be more costly to the mothers, but ensure a quicker development of the vulnerable larval stage, among other advantages, as proposed by Jolivet and Hawkeswood (1995) and Chaboo et al. (2014 and references therein), which is why it is sometimes considered to be a parental care preceding birth (Hinton 1981). Interestingly, viviparous species may result in solitary larvae, as in *P. axillaris* (Fig. 1–E2), or larval aggregations, as in *P. jucunda* (Fig. 1–P2). Oviparous species may also have solitary or gregarious larvae, as in *P. dejeani* (Fig. 1–I2) and *Z. appendiculata* (Fig. 1–Y2), respectively, although larval aggregations seem rarer in this type of reproduction. In their work on subsocial neotropical Doryphorini, Windsor et al. (2013) found, among *Platyphora* species, two with solitary larvae and nine which formed larval aggregations, but all eleven species were larviparous. We observed a single case of larval cannibalism in the viviparous *P. curticollis* during laboratory rearing (Fig. 1–H2), a behavior already described for some Chrysomelinae genera (Wade 1994, Mafra-Neto and Jolivet 1996, Windsor et al. 2013) that grants nutritional benefits.

Except for the eggs of *Z. appendiculata*, which are laid in masses on the underside of its host plant leaves (Fig. 1–Y5), no other eggs of oviparous Chrysomelinae were found in the field. This is probably because chrysomelids often lay their eggs in the soil or in secluded parts of plants (Selman 1994). All oviparous species reared in laboratory laid chorion-covered yellowish eggs on the bottom of the vials or on leaves, normally grouped in clutches (Fig. 1–W2). In the field, the number of eggs of *Z. appendiculata* varied from 80 to 100 per group (90.4 ± 8.3 SD; n=7 egg masses), and larval aggregations comprised between 10 and 233 individuals per group (49.2 ± 38.7; n=50 groups). Larvae of different egg masses may cohabit the same aggregation of this species, since differently sized larvae were observed in the same aggregation. *Platyphora fraterna* larvae (Fig. 1–N2) were grouped in aggregations of 24.9 ± 13.1 SD individuals (n=14 groups), with a minimum of seven and maximum of 44 larvae per group. No pupa has yet been found in the field, but in the laboratory, prepupae always buried themselves in earth layer at the bottom of the vials. While pupation in Chrysomelinae may be arboreal or underground (Takizawa 1976), it seems that underground pupation is most common in our taxa as indicated by laboratory rearing.

Only a few observations on natural enemies of Chrysomelinae were made. Phoretic wasps of Pteromalidae (Hymenoptera) were found on adults of *Grammodesma rubroaenea* (Fig. 1–D1) and *Deuterocampta sedula* (Fig. 1–B1). Pteromalidae are well known parasitoids of chrysomeline larvae (Cox 1994). On one occasion, a *Podisus* (Hemiptera) nymph was seen preying on a larva of *Z. appendiculata* (Fig. 1–Y6). Many chrysomeline species presented unprotected larvae without any apparent behavioral defense, but several gain chemical defenses by the sequestration of host plants toxins or by synthesizing defensive compounds from plant precursors, especially in *Platyphora* (Pasteels et al. 2001, Termonia et al. 2002).

## Conclusions

The high proportion of new host, biological data and occurrence records in Rio de Janeiro reflects the limited knowledge we have about this subfamily in this immediate area. In Brazil, the picture is not very different, as no inventory for the subfamily has been compiled and the relatively few published records come from ecological studies such as Medeiros and Vasconcellos-Neto (1994), Medeiros et al. (1996), Macedo et al. (1998), Vasconcellos-Neto and Jolivet (1998), and Flinte et al. (2009b, 2015). However, Chrysomelinae is known to be very species-rich in Brazil, including known endemic species, such as *Elythrosphaera lahtivirtai* (Macedo et al. 1998). Because of their high host specificity (Jolivet 1988, Jolivet and Hawkeswood 1995) and low dispersal ability (Freijeiro and Baselga 2016) the chrysomelines are expected to have many narrowly distributed species, especially in mountainous areas, as has already been found for other tropical Chrysomelidae species (García-Robledo et al. 2016, Macedo et al. 2016). These traits then would make these Brazil species especially vulnerable to extinction as the mountains within the Atlantic forest biome are largely degraded and threatened (Martinelli 2007).

## References

[B1] Bechyně J (1954) Beiträge zur Kenntnis der echten Chrysomeliden (Col. Phytophaga). Entomologische Arbeiten aus dem Museum G. Frey 5: 581–674.

[B2] Bechyně J (1958) Notizen zu den neotropischen Chrysomeloidea (Col. Phytophaga). Entomologische Arbeiten aus dem Museum G. Frey 9: 478–706.

[B3] Bechyně J Springlová de Bechyně B (1965) Notes sur les Chrysomelidae s. str. de Venezuela et des pays limitrophes. Revista de la Facultad de Agronomía (Maracay) 3: 44–110.

[B4] Bechynӗ J (1980) El Jannelismo y la evolucion. Grafindustrial, Maracay, 181 pp.

[B5] Bernardi N Scivittaro A (1991) Estágios imaturos de *Platyphora zonata* (Germar, 1824) (Coleoptera, Chrysomelidae, Chrysomelinae). Revista Brasileira de Zoologia 7(4): 531–534. https://doi.org/10.1590/S0101-81751990000400011

[B6] Blackwelder RE (1944) Checklist of the coleopterous insects of Mexico, Central America, The West Indies, and South America. Part 4. U.S. National Museum, Bulletin 185. Smithsonian Institution, Washington, D.C., 763 pp.

[B7] Bontems C (1988) Localization of spermatozoa inside viviparous and oviparous females of Chrysomelinae. In: Jolivet P Petitpierre E Hsiao TH (Eds) Biology of Chrysomelidae. Kluwer Academic Publishers, Dordrecht, 299–315. https://doi.org/10.1007/978-94-009-3105-3_18

[B8] Burgos-Solorio A Anaya-Rosales S (2004) Los Crisomelinos (Coleoptera: Chrysomelidae: Chrysomelinae) del Estado de Morelos. Acta Zoológica Mexicana (n.s. ) 20: 39–66. http://www.scielo.org.mx/scielo.php?script=sci_arttext&pid=S0065-17372004000300004&lng=es&nrm=iso

[B9] Chaboo CS Flowers RW (2015) Beetles (Coleoptera) of Peru: A survey of the families. Chrysomelidae: Chrysomelinae. Journal of the Kansas Entomological Society 88: 380–383. https://doi.org/10.2317/kent-88-03-380-383.1

[B10] Chaboo CS Frieiro-Costa FA Gómez-Zurita J Westerduijn R (2014) Origins and diversifcation of subsociality in leaf beetles (Coleoptera: Chrysomelidae: Cassidinae: Chrysomelinae). Journal of Natural History 48: 2325–2367. https://doi.org/10.1080/00222933.2014.909060

[B11] Cox ML (1994) The Hymenoptera and Diptera parasitoids of Chrysomelidae. In: Jolivet P Cox ML Petitpierre E (Eds) Novel Aspects of the Biology of Chrysomelidae. Kluwer Academic Publishers, Dordrecht, 419–467. https://doi.org/10.1007/978-94-011-1781-4_35

[B12] Daccordi M (1994) Notes for phylogenetic study of Chrysomelinae, with descriptions of new taxa and a list of all known genera (Coleoptera: Chrysomelidae, Chrysomelinae). In: Furth DG (Ed.) Proceedings of the Third International Symposium on the Chrysomelidae. Backhuys Publishers, Leiden, 60–84.

[B13] Daccordi M (1996) Notes on the distribution of the Chrysomelinae and their possible origin. In: Jolivet P Cox ML (Eds) Chrysomelidae Biology, volume 1: Classification, Phylogeny and Genetics. SPB Academic Publishing, Amsterdam, 399–412.

[B14] Dury GJ Bede JC Windsor DM (2014) Preemptive Circular Defence of Immature Insects: Definition and occurrence of Cycloalexy Revisited. Psyche 2014: Article ID 642908, 13 pp. https://doi.org/10.1155/2014/642908

[B15] Flinte V Borowiec L Freitas S Viana JH Fernandes FR Nogueira-de-Sá F Macedo MV Monteiro RF (2009a) Tortoise beetles of the State of Rio de Janeiro, Brazil (Coleoptera: Chrysomelidae: Cassidinae). Genus - International Journal of Invertebrate Taxonomy 20: 571–614. http://www.cassidae.uni.wroc.pl/Flinte_Cassidinae%20Rio%20Janeiro.pdf

[B16] Flinte V Freitas S Macedo MV Monteiro RF (2011) Altitudinal and temporal distribution of *Plagiometriona* Spaeth, 1899 (Coleoptera, Chrysomelidae, Cassidinae) in a tropical forest in southeast Brazil. ZooKeys 157: 15–31. https://doi.org/10.3897/zookeys.157.1179 10.3897/zookeys.157.1179 PMC3253640 22303101

[B17] Flinte V Hentz E Morgado BM Lima ACM Khattar G Monteiro RF Macedo MV (2015) Biology and phenology of three leaf beetle species (Chrysomelidae) in a montane forest in southeast Brazil. In: Jolivet P Santiago-Blay J Schmitt M (Eds) Research on Chrysomelidae 5. ZooKeys 547: 119–132. https://doi.org/10.3897/zookeys.547.9015 10.3897/zookeys.547.9015 PMC4714337 26798318

[B18] Flinte V Macedo MV Monteiro RF (2008) Tortoise beetles (Chrysomelidae: Cassidinae) of a tropical rain forest in Rio de Janeiro, Brazil. In: Jolivet P Santiago-Blay J Schmitt M (Eds) Research on Chrysomelidae, vol. 1. Brill, Leiden, 194–209.

[B19] Flinte V Macedo MV Monteiro RF (2009b) Chrysomelids and their host plants along an altitudinal gradient in a tropical Atlantic Rain Forest in Rio de Janeiro, Brazil. In: Jolivet P Santiago-Blay J Schmitt M (Eds) Research on Chrysomelidae, vol. 2. Brill, Leiden, 31–56. https://doi.org/10.1163/ej.9789004169470.1-299.14

[B20] Flowers RW (2004) The genera of Chrysomelinae (Coleoptera: Chrysomelidae) in Costa Rica. Revista de Biología Tropical 52: 77–83. https://doi.org/10.15517/rbt.v52i1.14754 10.15517/rbt.v52i1.14754 17357402

[B21] Freijeiro A Baselga A (2016) Spatial and environmental correlates of species richness and turnover patterns in European cryptocephaline and chrysomeline beetles. In: Jolivet P Santiago-Blay J Schmitt M (Eds) Research on Chrysomelidae 6. ZooKeys 597: 81–99. https://doi.org/10.3897/zookeys.597.6792 10.3897/zookeys.597.6792 PMC4926622 27408587

[B22] García-Robledo C Kuprewicz EK Staines CL Erwin TL Kress WJ (2016) Limited tolerance by insects to high temperatures across tropical elevational gradients and the implications of global warming for extinction. Proceedings of the National Academy of Sciences 113: 680–685. https://doi.org/10.1073/pnas.1507681113 10.1073/pnas.1507681113 PMC4725502 26729867

[B23] Grégoire JC (1988) Larval gregariousness in the Chrysomelidae. In: Jolivet P Petitpierre E Hsiao TH (Eds) Biology of Chrysomelidae. Kluwer Academic Publishers, Dordrecht, 253–260. https://doi.org/10.1007/978-94-009-3105-3_15

[B24] Grissell EE de Santis L (1987) A new species of *Erixestus* (Hymenoptera: Pteromalidae), an egg parasitoid of *Calligrapha polyspila* (Coleoptera: Chrysomelidae) in Argentina. Proceedings of the Entomological Society of Washington 89(2): 264–268.

[B25] Hinton HE (1981) Biology of Insect Eggs. Vol 1. Pergamon Press, Elmsford, 473 pp.

[B26] Jolivet P (1988) Food habits and food selection of Chrysomelidae. Bionomic and evolutionary perspectives. In: Jolivet P Petitpierre E Hsiao TH (Eds) Biology of Chrysomelidae. Kluwer Academic Publishers, Dordrecht, 1–24. https://doi.org/10.1007/978-94-009-3105-3_1

[B27] Jolivet P (1997) Biologie des Coléoptères Chrysomélides. Société nouvelle des éditions Boubée, Paris, 280 pp.

[B28] Jolivet P Hawkeswood TJ (1995) Host-plants of Chrysomelidae beetles of the world: an essay about the relationships between leaf beetles and their food-plants. Backhuys Publishers, Leiden, 281 pp.

[B29] Jolivet P Vasconcellos-Neto J Weinstein P (1990) Cycloalexy: a new concept in the larval defense of insects. Insecta Mundi 4(1-4): 133–142. http://digitalcommons.unl.edu/insectamundi/394

[B30] Macedo MV Flinte V Araujo CO Silveira LFL Bouzan AM Dufrayer R Viana JH Araujo RO Hentz E Monteiro RF (2016) Elevational ranges and local extinction risk of beetles occurring in the “Campos de Altitude” in southeastern Brazil. Oecologia Australis 20(2): 121–132. https://doi.org/10.4257/oeco.2016.2002.09

[B31] Macedo MV Vasconcellos-Neto J Jolivet P (1998) New Biological data on the apterous beetle *Elytrosphaera lahtivirtai* Bechyně Chrysomelidae, Chrysomelinae) and remarks on the biology and distribution of the genus. In: Biondi M Daccordi M Furth DG (Eds) Proceedings of the Fourth International Symposium on the Chrysomelidae. Museo Regionale di Scienze Naturali, Torino, 271–279.

[B32] Mafra-Neto A Jolivet P (1996) Cannibalism in leaf beetles. In: Jolivet P Cox ML (Eds) Chrysomelidae Biology, Volume 2: Ecological studies. SPB Academic Publishing, Amsterdam, 195–211.

[B33] Martinelli G (2007) Mountain biodiversity in Brazil. Brazilian Journal of Botany 30: 587–597. https://doi.org/10.1590/S0100-84042007000400005

[B34] Medeiros L Ferro DN Mafra-Neto A (1996) Association of Chrysomelid beetles with solanaceous plants in the south of Brazil. In: Jolivet P Cox ML (Eds) Chrysomelidae Biology, Volume 2: Ecological studies. SPB Academic Publishing, Amsterdam, 339–364.

[B35] Medeiros L Vasconcellos-Neto J (1994) Host plant and seasonal abundance patterns of some Brazilian Chrysomelidae. In: Jolivet P Cox ML Petitpierre E (Eds) Novel aspects of the biology of Chrysomelidae. Kluwer Academic Publishers, Dordrecht, 185–189. https://doi.org/10.1007/978-94-011-1781-4_11

[B36] Nogueira-de-Sá F Medeiros L Macedo MV (2004) Phenology of populations of tortoise beetles (Cassidinae) in Brazil. In: Jolivet P Santiago-Blay JA Schmitt M (Eds) New Developments in the Biology of Chrysomelidae. SPB Academic Publishing, The Hague, 647–658.

[B37] Olckers T (1998) Biology and host range of *Platyphora semiviridis*, a leaf beetle evaluated as a potential biological control agent for *Solanum mauritianum* in South Africa. BioControl 43: 225–239. https://doi.org/10.1023/A:1009912011121

[B38] Olckers T (2000) Biology and physiological host range of four species of *Platyphora* Gistel (Coleoptera: Chrysomelidae) associated with *Solanum mauritianum* Scop. (Solanaceae) in South America. The Coleopterists Bulletin 54(4): 497–510. https://doi.org/10.1649/0010-065X(2000)054[0497:BAPHRO]2.0.CO;2

[B39] Pasteels JM Termonia A Windsor DM Witte L Teuring C Hartmann T (2001) Pyrrolizidine alkaloids and pentacyclic triterpene saponins in the defensive secretions of *Platyphora* leaf beetles. Chemoecology 11: 113–120. https://doi.org/10.1007/PL00001840

[B40] Reid CAM (1995) A cladistic analysis of subfamilial relationships in the Chrysomelidae *sensu lato* (Chrysomeloidea). In: Pakaluk J Slipinski SA (Eds) Biology, Phylogeny, and Classification of Coleoptera: papers celebrating the 80th birthday of Roy A. Crowson. Muzeum i Instytut Zoologii PAN, Warszawa, 559–631.

[B41] Reid CAM (2006) A taxonomic revision of the Australian Chrysomelinae, with a key to the genera (Coleoptera: Chrysomelidae). Zootaxa 1292: 1–119.

[B42] Riley EG Clark SM Flowers RW Gilbert AJ (2002) Family 124. Chrysomelidae Latreille 1802. In: Arnett RH Thomas MC Skelley PE Frank JH (Eds) Chrysomelinae, in American Beetles, vol. 2. Polyphaga: Scarabaeoidea through Curculionoidea. CRC Press, Boca Raton, 648–653.

[B43] Sampaio AA Monné ML (2016) Inventário das espécies de *Grammodesma* Achard, 1923 (Coleoptera: Chrysomelidae: Chrysomelinae) ocorrentes no Parque Nacional do Itatiaia, Sudeste do Brasil. In: Da-Silva ER Passos MIS Aguiar VM Lessa CSS Coelho LBN (Eds) III Simpósio de Entomologia do Rio de Janeiro, Rio de Janeiro (Brasil), setembro 2015. Perse Editora, Rio de Janeiro, 56–61. https://doi.org/10.13140/RG.2.1.1474.2649

[B44] Seeno TN Wilcox JA (1982) Leaf Beetle Genera (Coleoptera: Chrysomelidae). Entomography 1: 1–221.

[B45] Selman BJ (1994) Eggs and oviposition in chrysomelid beetles. In: Jolivet PH Cox ML Petitpierre E (Eds) Novel aspects of the biology of Chrysomelidae. Kluwer Academic Publishers, Dordrecht, 69–74. https://doi.org/10.1007/978-94-011-1781-4_2

[B46] Takizawa H (1976) Larvae of the genus *Gonioctena* Chevrolat (Coleoptera: Chrysomelidae): descriptions of Japanese species and the implications of larval characters for the phylogeny. Kontyû 44: 444–468

[B47] Termonia A Pasteels JM Windsor DM Milinkovitch MC (2002) Dual chemical sequestration: a key mechanism in transitions among ecological specialization. Proceedings of the Royal Society B 269(1486): 1–6. https://doi.org/10.1098/rspb.2001.1859 11788029 10.1098/rspb.2001.1859 PMC1690864

[B48] Vasconcellos-Neto J Jolivet P (1994) Cycloalexy among chrysomelid larvae. In: Jolivet P Cox ML Petitpierre E (Eds) Novel Aspects of the Biology of Chrysomelidae. Kluwer Academic Publishers, Dordrecht, 303–309. https://doi.org/10.1007/978-94-011-1781-4_23

[B49] Vasconcellos-Neto J Jolivet P (1998) Are Brazilian species of *Elytrosphaera* (Col. Chrysomelidae), an apterous genus, threatened of extintion? In: Biondi M Daccordi M Furth DG (Eds) Proceedings of the Fourth International Symposium on the Chrysomelidae. Museo Regionale di Scienze Naturali, Torino, 299–309.

[B50] Veloso HP Rangel-Filho ALR Lima JCA (1991) Classificação da Vegetação Brasileira Adaptada a Um Sistema Universal. Fundação Instituto Brasileiro de Geografia e Estatística (IBGE), Rio de Janeiro, 124 pp.

[B51] Wade MJ (1994) The biology of the imported willow leaf beetle *Plagiodera versicolora* (Laicharting). In: Jolivet PH Cox ML Petitpierre E (Eds) Novel aspects of the biology of Chrysomelidae. Kluwer Academic Publishers, Dordrecht, 541–547. https://doi.org/10.1007/978-94-011-1781-4_41

[B52] Windsor DM Dury GJ Frieiro-Costa FA Lanckowsky S Pasteels JM (2013) Subsocial Neotropical Doryphorini (Chrysomelidae, Chrysomelinae): new observations on behavior, host plants and systematics. In: Jolivet PH Santiago-Blay J Schmitt M (Eds) Research on Chrysomelidae 4. ZooKeys 332: 71–93. https://doi.org/10.3897/zookeys.332.5199 10.3897/zookeys.332.5199 PMC3805320 24163582

